# CMTM3 Inhibits Human Testicular Cancer Cell Growth through Inducing Cell-Cycle Arrest and Apoptosis

**DOI:** 10.1371/journal.pone.0088965

**Published:** 2014-02-28

**Authors:** Zesong Li, Jun Xie, Jianting Wu, Wenjie Li, Liping Nie, Xiaojuan Sun, Aifa Tang, Xianxin Li, Ren Liu, Hongbing Mei, Feng Wang, Zhiping Wang, Yaoting Gui, Zhiming Cai

**Affiliations:** 1 Shenzhen Key Laboratory of Genitourinary Tumor, Shenzhen Second People's Hospital, First Affiliated Hospital of Shenzhen University, Shenzhen, China; 2 Guandong Key Laboratory of Male Reproductive Medicine and Genetics, Peking University Shenzhen Hospital, Shenzhen PKU-HKUST Medical Center, Shenzhen, China; 3 Department of Clinical Laboratory, Peking University Shenzhen Hospital, Shenzhen, China; 4 Department of Urology, Suzhou municipal Hospital, Suzhou, Anhui, China; 5 Department of Urology, The Second Hospital of Lanzhou University, Lanzhou, China; German Cancer Research Center, Germany

## Abstract

Human CMTM3 has been proposed as a putative tumor suppressor gene. The loss of CMTM3 has been found in several carcinomas. However, the regulation of CMTM3 expression and its function in tumor progression remain largely unknown. Here, we investigated the regulation of CMTM3 expression, function and molecular mechanism in human testicular cancer cells. CMTM3 was frequently downregulated or silenced in testicular cancer cell lines and tumor tissues but highly expressed in normal testis tissues. The re-expression of CMTM3 significantly suppressed the colony formation, proliferation, and migration capacity of testicular cancer cells by inducing a G2 cell cycle arrest and apoptosis. Moreover, the re-expression of CMTM3 activated the transcription of p53, induced p53 accumulation, up-regulated the expression of p21, and increased the cleavage of caspase 9, 8, 3, and PARP. The downregulation of *CMTM3* in clinical tumor tissues was associated with the methylation of a single CpG site located within the Sp1/Sp3-responsive region of the core promoter. These results indicate that CMTM3 can function as tumor suppressor through the induction of a G2 cell cycle arrest and apoptosis. CMTM3 is thus involved in testicular cancer pathogenesis, and it is frequently at least partially silenced by the methylation of a single, specific CpG site in tumor tissues.

## Introduction

Testicular germ cell tumors (TGCTs) are common in men aged 15 to 35 years and account for 1% of all malignant neoplasms in males [1]. The incidence of TGCTs has increased dramatically over the last century [2].

TGCTs originate from transformed gonocytes or undifferentiated spermatogonia, which are derived from fetal germ cells and adult germ stem cells, respectively. TGCTs are classified as seminomas or non-seminomatous germ cell tumors according to their histologic characteristics [1]. Seminomas are the most frequent (50–70%) testicular germ cell tumors. Non-seminomatous germ cell tumors include embryonal cell carcinoma, yolk sac tumors, choriocarcinoma, and teratomas [1]. TGCTs have become one of the most curable solid neoplasms, due to advances in diagnostic and therapeutic methods [3]. However, the molecular mechanisms operating in TGCTs are not fully understood.

CKLF-like MARVEL transmembrane domain containing 3 (CMTM3) belongs to the chemokine-like factor gene superfamily, a novel family that is similar to the chemokine and transmembrane 4 superfamilies of signaling molecules. *CMTM3* is one of several chemokine-like factor genes located in a cluster on chromosome 16q22. CMTM3 protein contains one leucine zipper and two LXXLL motifs. This 20-kDa protein is localized to the cytoplasm and serves as a scaffold for proteins in the endoplasmic reticulum and the nuclear membrane [4]. *CMTM3* is highly expressed in the male reproductive system, with the highest expression level in the testes [4].

Previous studies also indicated that several members of the CMTM superfamily may play important roles in the immune and male reproductive systems and in tumorigenesis [5]–[12]. It has recently been shown that *CMTM3* is silenced or down-regulated in gastric, breast, nasopharyngeal, esophageal, colon and renal carcinomas [8], [13]. Its expression was inversely correlated with its promoter CpG methylation status [8], [13]. The re-expression of CMTM3 in tumor cells lacking its expression leads to the suppression of cell growth and apoptosis, which suggests that CMTM3 is a novel tumor suppressor [8]. However, its role in cancer development and progression has not been clearly defined to date.

In this study, we observed that *CMTM3* was frequently down-regulated in testicular cancer tissues via methylation at a specific, single CpG site located within the Sp1/Sp3-responsive region of the promoter. The ectopic expression of *CMTM3* inhibited the colony formation, proliferation, migration and invasive capacity of testicular cancer cells. These functions of CMTM3 were achieved by modulating cell cycle progression and apoptosis.

## Materials and Methods

### Cancer cells and clinical samples

A human seminoma cell line (NCCIT), prostate cancer cell lines (LNCaP, DU145, PC3 and 22RV1), renal cancer cell lines (OSRC-2 and 786-O), bladder tumor cell lines (HTB9-5637 and T24) and a HEK-293 human epithelial kidney cell line were used. Cells were cultured in RPMI 1640 media (GIBCO/BRL) supplemented with 10% fetal bovine serum (FBS) at 37°C with an atmosphere of 5% CO_2_.

Twenty pairs of testicular cancer tissues and the matched non-cancerous testicular tissues were obtained from patients undergoing primary surgery at the Shenzhen Second People's Hospital and Peking University Shenzhen Hospital with patients' or guardians' written consent,and has been approved by the ethics committee of the Shenzhen Second People's Hospital, Shenzhen, China. Samples were either immediately fixed in 10% neutral formalin for histology and immunohistochemistry or snap frozen in liquid nitrogen and stored at −80°C for further PCR. H&E-stained slides from all cases were reviewed to confirm the diagnoses. The testicular germ cell tumors consisted of 15 cases of seminoma, 2 embryonal carcinoma, 1 teratoma, 1 mixed embryonal carcinoma and teratoma, and 1 Leydig cell tumor according to World Health Organization criteria [14]. Five normal testicular tissue samples from adults who had undergone routine autopsy were also used for controls. The patients are 6–58 years old with an average of 38 years.

### Semiquantitative reverse transcription-PCR and real-time PCR

The isolation of total RNA and reverse transcription were performed as previously described [15]. The semi-quantitative PCR analysis of *CMTM3* was performed using specific primers, and *GAPDH* was used as the internal control. *CMTM3* and *GAPDH* primers were *CMTM3*-F: 5′-TTTTATCTGCTATGTGGCGTCC-3′, and *CMTM3*-R: 5′-TGTCTTGTGGGCTGTGGTCTC-3′; *GAPDH*-F: 5′-GTCAACGGATTTGGTCGTATTG-3′, and *GAPDH*-R: 5′-CTCCTGGAAGATGGTGATGGG-3′. Real-time PCR assays were performed using Platinum SYBR Green qPCR Supermix UDG (Invitrogen Corporation, Carlsbad, CA). The assay was conducted in triplicate using different primer sets: *CMTM3*-F: 5′-GCTTCTTTGCTACCATCGTGTTTG-3′, and *CMTM3*-R: 5′-GCCTTCAGTCAG AGTCCGAGTC-3′; *GAPDH*-F: 5′-CGCTCTCTGCTCCTCCTGTTC -3′; *GAPDH*-R: 5′-ATCCGTTGACTCCGACCTTCAC-3′. The relative expression level of *CMTM3* was determined by using the 2^−ΔΔCt^ method [16].

### Protein extraction and Western blot analysis

Tissues or cells were lysed by RIPA buffer (Sigma-Aldrich, St. Louis, MO, USA) supplemented with a protease inhibitor cocktail and phosphatase inhibitors. A quantity of 30 µg of total protein was resolved on SDS-12% polyacrylamide gels and transferred to PVDF membranes (Hybond-P, Amersham Biosciences Piscataway, NJ, USA). After blocking with 5% BSA in tris-buffered saline with 0.1% Tween 20 (TBST) at room temperature for 2 h, the membrane was probed with primary antibody at 4°C overnight. After washing three times with TBST buffer, membranes were incubated with horseradish peroxidase-conjugated IgG. The blotting signals were visualized with the Super Signal West Pico Chemiluminescent substrate (Pierce, Rockford, IL, USA). Membranes were stripped with stripping buffer at 40°C for 30 min with gentle shaking and reprobed with a rabbit polyclonal antibody against β-actin (1∶10000, Santa Cruz Biotechnology) as a control for protein loading.

### Construction of promoter reporter plasmids, transfection and luciferase assay

To construct a series of *CMTM3* promoter-driven luciferase reporter plasmids, six DNA fragments of the upstream of *CMTM3* start codon were amplified by PCR (PCR Primers in Table S1 in File S1) and cloned into the pGL3-Basic vector (Promega). The insertions were confirmed by direct sequencing. 293FT and PC3 cells were plated in 24-well culture plates at 1×10^5^ cells/well and transiently transfected with the pGL3 promoter reporter and pRLSV40 as described previously [15], [17]. When indicated, pCMV-Sp1 or pCMV-Sp3 expression plasmids (OriGene, Rockville, MD, USA) were co-transfected into the cells. Luciferase activity was determined using the Dual-Luciferase Reporter Assay System (Promega, Madison, WI) and normalized to the Renilla luciferase activity. Each experiment was performed three times in triplicate. Activity was defined as the ratio of firefly luciferase to Renilla luciferase. For the *in vitro* methylation assay on the *CMTM3* promoter fragment, Sss I methylase was used as reported previously [15].

### DNA bisulfite treatment and methylation analysis

The bisulfite modification of DNA and bisulfite genomic sequencing (BGS) were performed as described previously [15], [18]. Bisulfite sequencing PCR primers (BGS-F, TAGATAGTTTTTTTGGATAGGGGTAGA, and BGS-R, ACCTTTAAAAAAACAAAAAAAAACCC) were designed using the online program, MethPrimer (http://itsa.ucsf.edu/urolab/MethPrimer) [19]. PCR was performed for 40 cycles with Hotstart Taq polymerase (Qiagen, Hilden, Germany). PCR products were cloned into the pGEM-T (Promega, Madison, WI) vector. Eight to ten white clones for each sample were randomly selected for sequencing.

### Immunohistochemistry

The immunohistochemical analysis of CMTM3 was performed using a standard two-step technique as described previously [15]. After deparaffinization and rehydration, tissue slides were cooked to retrieve the antigen in a microwave oven with 10 mM citrate buffer (pH 6) for 15 min. The slides were immersed in 3% H_2_O_2_ for 20 min to block endogenous peroxidase activity, washed with PBS, and incubated with 3% normal bovine serum in TBS for 30 min to prevent the non-specific binding of the primary antibody. Then, tissue slides were incubated with a purified rabbit polyclonal antibody against CMTM3 (1∶500, kindly provided by Dr. Han, Peking University Center for Human Disease Genomics) or normal rabbit IgG as a control at 4°C overnight. After washing with PBS three times, the slides were incubated with goat anti-rabbit IgG-HRP (sc-2030, Santa Cruz, CA, USA). After washing, tissues were stained with freshly prepared DAB solution (DAKO, Carpinteria, CA) and visualized and photographed with a Leica DM4000B (Leica Microsystems, Inc.).

The immunohistochemical expression of CMTM3 was evaluated based a representative high power field from 2 tissue array spots by two independent investigators. The expression of CMTM3, based on calculating a total immunostaining score (TIS) as the product of a proportion score (PS) and an intensity score (IS), was quantified under microscope and classified into four subgroups: no expression (0–4); weak expression (+:5,6,8); moderate expression (++: 9,10,12); intense expression (+++: 15) [20].

### Adenoviral infection

The adenovirus carrying the *CMTM3* gene (Ad-*CMTM3*) and the empty adenovirus (Ad-null) were kindly provided by Dr. Han's Lab [7]. NCCIT cells were seeded at 5000 cells/cm^2^ into tissue culture plates, and after 24 h, infection was accomplished by exposing the cells to adenovirus at the required multiplicity of infection (MOI) in serum-free cell culture media for 60 min, followed by the addition of serum-containing media for 1–4 days.

### Cell proliferation

For the analysis of cell proliferation, NCCIT cells were infected as above, and cell proliferation was measured at 24, 48 and 72 h using the Cell Counting Kit-8 (CCK-8) following the manufacturer's instruction. The spectrophotometric absorbance at 490 nm was measured using a microplate reader. The data are reported as the mean of at least three independent experiments.

### Colony formation assay

Cells were infected with Ad-*CMTM3* or Ad-null. 24 h after infection, 1000 cells were plated into each well of 6-well plates and kept in complete media for 2 weeks. Surviving colonies (≥50 cells per colony) were fixed with methanol, stained with crystal violet, counted and photographed. Each experiment was run in triplicate and performed three times.

### Wound-healing assay

For the in vitro wound-healing assay, cells infected with Ad-*CMTM3* or Ad-null were cultured in six-well plates until confluent. A straight scratch was created across the cell layer using a sterile micropipette tip, and the debris was removed by washing the cells with serum-free media. Cells were photographed 0 and 36 h after wounding. The experiments were performed in triplicate.

### Apoptosis assays

Apoptotic cell death was assessed by flow cytometry using the Annexin V–FITC/PI Apoptosis Detection Kit according to the manufacturer's instructions. After 72 h, cells were harvested and incubated with Annexin V-FITC and PI for 30 min in the dark at 4°C. Flow cytometric analysis was immediately performed. The data are presented as bi-parametric dot plots showing Annexin V-FITC green fluorescence versus PI red fluorescence. The experiments were performed three times.

### Quantitative PCR for apoptosis pathway

Forty-eight hours after cells were infected with Ad-*CMTM3* or Ad-null, cells were harvested, and total RNA was isolated and purified with the RNeasy RNA Isolation Kit (Qiagen). A quantity of 2 µg of total RNA was used to synthesize cDNAs with the RT2 First Strand Kit (SABiosciences, QIAGEN). The cDNAs were added to a 96-well plate from the RT^2^ Profiler PCR array system (human apoptosis PCR array). Quantitative RT-PCR for 84 genes related to human apoptosis was performed using the iCYCLER iQ5 Real-Time PCR System (Bio-Rad, Hercules, CA). Data analysis was performed using the ΔΔ*C_T_* method with five housekeeping genes (*GAPDH*, *RPL13A*, *B2M*, *ACTB*, and *HPRT1*) selected for normalization. Transcripts were considered absent if C_t_>35 and were removed from analysis. Changes in gene expression were determined by comparing gene transcription levels in cells infected with Ad-CMTM3 to cells infected with Ad-null.

### Cell cycle analysis

Forty-eight hours after cells were infected with Ad-*CMTM3* or Ad-null, the cells were trypsinized, washed twice, resuspended in phosphate-buffered saline (PBS), and fixed in ice-cold 70% ethanol. After the cells were stained with propidium iodide (PI), cell cycle analysis was performed by flow cytometry on a fluorescence-activated cell sorting scanner. The cell cycle distribution was analyzed with Cell Quest software.

### Statistical analyses

Statistical analyses were performed using SPSS version 19 software. The expression analysis of CMTM3 between testicular cancer tissues and the corresponding adjacent tissues were assessed using Student's t-test. The comparisons of categorical variables were performed using the x^2^ test or 2-tailed t-test. Differences were considered statistically significant if a p value was less than 0.05.

## Results

### CMTM3 is frequently downregulated in testicular cancers

**Figure 1:**
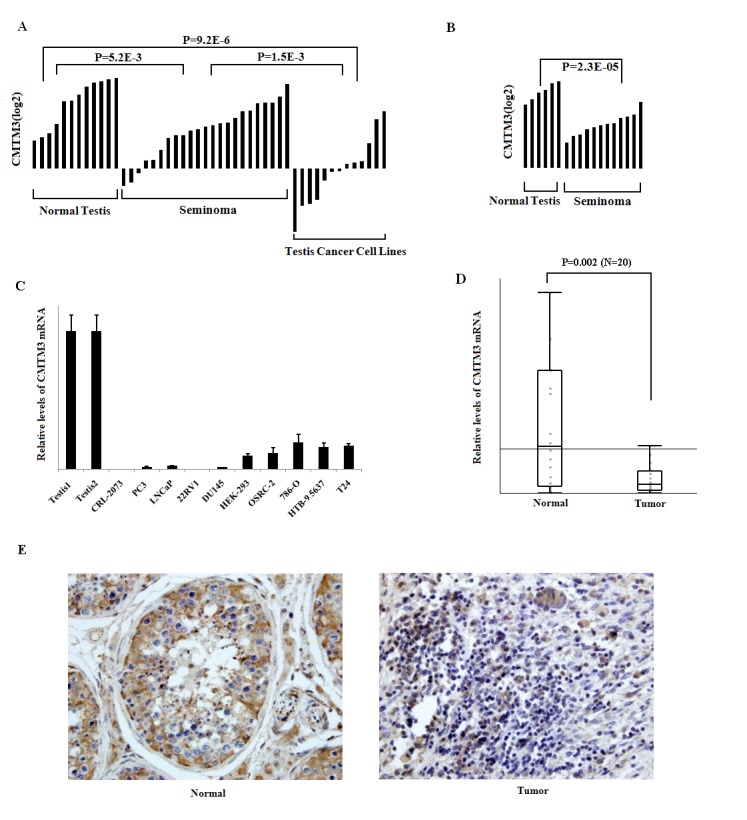
CMTM3 expression is decreased in testicular tumors. (A and B) The expression of CMTM3 in individual samples from normal testis tissues or testicular cancer is shown (data normalized to the log2 scale from the Sperger datasets [23] and Korkola [22]). Comparisons between groups were performed using Student's t test, with P values indicated in each panel. (C) The expression of *CMTM3* in normal testis tissues and urogenital cancer cell lines were determined by real-time RT-PCR. Data are shown as the mean ± S.D. for three independent experiments. (D) The expression of *CMTM*3 in 20 testicular tumor specimens and paired normal testis tissues was determined by real-time RT-PCR. Data are shown as the mean ± S.D. from three independent experiments. (E) Representative immunohistochemistry staining for CMTM3 in testicular tumor specimens and paired normal testis tissues.

To investigate CMTM3's role in urogenital cancer, we first queried the Oncomine database [21] to assess the relative expression levels of *CMTM3* in urogenital cancer. Two independent studies showed that *CMTM3* expression was statistically significantly decreased in testicular tumors [22], [23] (Figure 1A, 1B). We also measured *CMTM3* mRNA levels in 10 urogenital cancer cell lines. Compared with the high expression of *CMTM3* in normal human testis tissues, the expression of *CMTM3* was silenced in a seminoma cell line (NCCIT) and down-regulated or silenced in 2 of 4 prostate cancer cell lines, 2 of 3 renal cancer cell lines and in none of the bladder tumor cell lines studied (Figure 1C). We further analyzed the expression of *CMTM3* in 20 paired testis tumor tissues and the corresponding benign adjacent tissues. *CMTM3* mRNA levels were silenced or strongly downregulated in 16 of 20 testis cancer cases with an overall 5-fold decrease compared to the corresponding benign adjacent tissue (*P* = 0.002) (Figure 1D). The results were confirmed in protein levels by immunohistochemistry staining (Figure 1E).

Furthermore, we extended the analysis of CMTM3 expression to an independent cohort of testis tissue microarray by immunohistochemistry (Table 1, Figure S1 in File S1). Of 75 cases of testis tumors, 36 (48%) cases of tumors showed no CMTM3 expression, 27 (36%) cases of tumors had low CMTM3 expression, and 12 (16%) cases of tumor had moderate CMTM3 expression. All of 18 (100%) cases of cancer adjacent normal tissues and normal testicular tissues had intensive or strong intensive CMTM3 expression (Table 1). All of 8 (100%) case of atrophy had intensive CMTM3 expression. Importantly, no single case was observed where CMTM3 staining was more intense in tumor than in normal testis, further highlighting the specificity of the observed pattern (Table 1). We also investigated the correlation between CMTM3 expressions in seminoma with pathological parameters, and noticed that decreased CMTM3 is significantly associated with advanced T stage (Table S2 in File S1).

**Table 1:** The expression of CMTM3 in testicular tissues and a series of testis tumors.

Groups		N = 101	CMTM3 protein levels			
			0	+	++	+++
Testicular cancer tissues	Seminoma	46	25	12	9	0
	Yolk sac tumor	8	4	3	1	0
	Embryonal carcinoma	16	5	9	2	0
	Teratoma	5	2	3	0	0
Testicular tissues	Atrophy	8	0	0	0	8
	Cancer adjacent normal tissue	13	0	0	0	13
	Normal testicular tissue	5	0	0	0	5

### Re-expression of CMTM3 inhibits cell growth and cell migration

**Figure 2:**
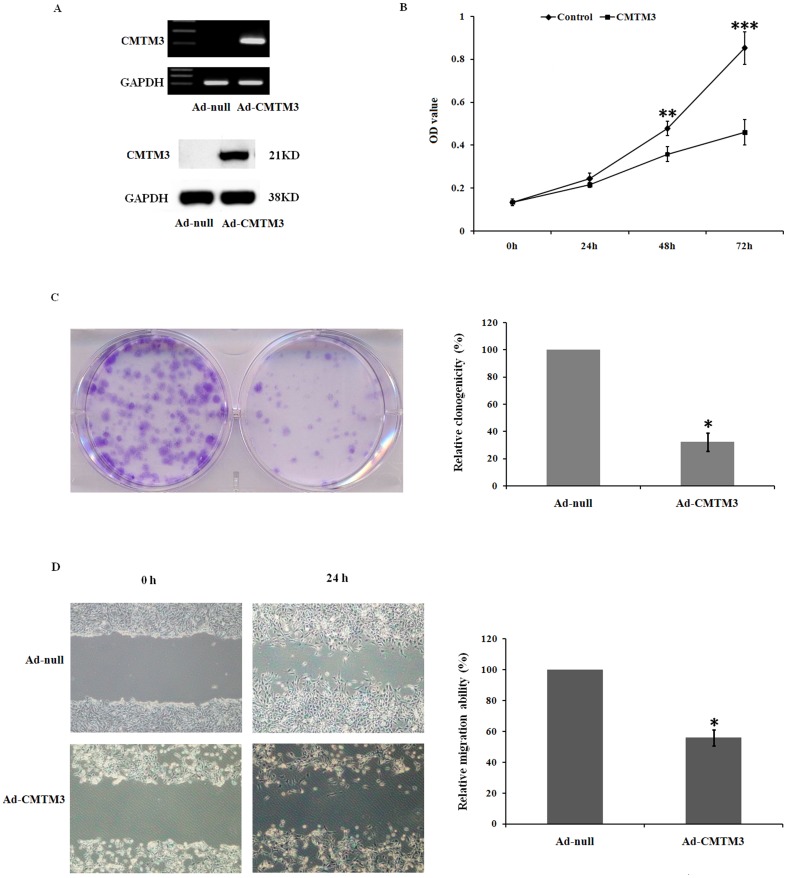
Re-expression of CMTM3 inhibited proliferation, colony formation and migration of testicular cancer cells. (A) Expression of CMTM3 in NCCIT cells after infection with Ad-CMTM3 was confirmed using RT-PCR and Western blots. (B) Cell proliferation was detected by the WST-1 assay in NCCIT cells following infection with Ad-null or Ad-*CMTM3*. (C) Representative results of colony-formation assays with NCCIT cells. (D) Migration was determined by a wound-healing assay in NCCIT cells infected with Ad-null or Ad-*CMTM3*. The results are representative of three independent experiments.

The silencing of *CMTM3* in testicular cancer indicated that CMTM3 might be a functional tumor suppressor in testicular carcinogenesis. To investigate the growth-inhibitory effect of *CMTM3*, the re-expression of *CMTM3* in the transiently infected NCCIT cells was confirmed by RT-PCR and Western blotting (Figure 2A). As shown in Figure 2B, cell growth was significantly decreased in Ad-*CMTM3*-infected NCCIT cells compared with Ad-null-infected control cells. The suppressive effect on cancer cell growth was further confirmed by a colony formation assay. Colony numbers were significantly reduced compared with the control cells to 32% in NCCIT cells infected with Ad-*CMTM3* (P<0.01) (Figure 2C).

In addition, the effect of CMTM3 on testicular cancer cell motility was examined by a wound-healing assay. Confluent monolayers of cells were scratched to form wounds and then cultured for 24 h. Ectopic CMTM3 expression led to significantly reduced wound healing cell migration compared to Ad-null-infected cells (Figure 2D). Ad-*CMTM3*-transfected cells became round and detached, whereas no obvious change was observed in Ad-null-infected cells, which is consistent with a previous report [8].

### Re-expression of CMTM3 induces cell cycle arrest in G2 phage and cell apoptosis

**Figure 3:**
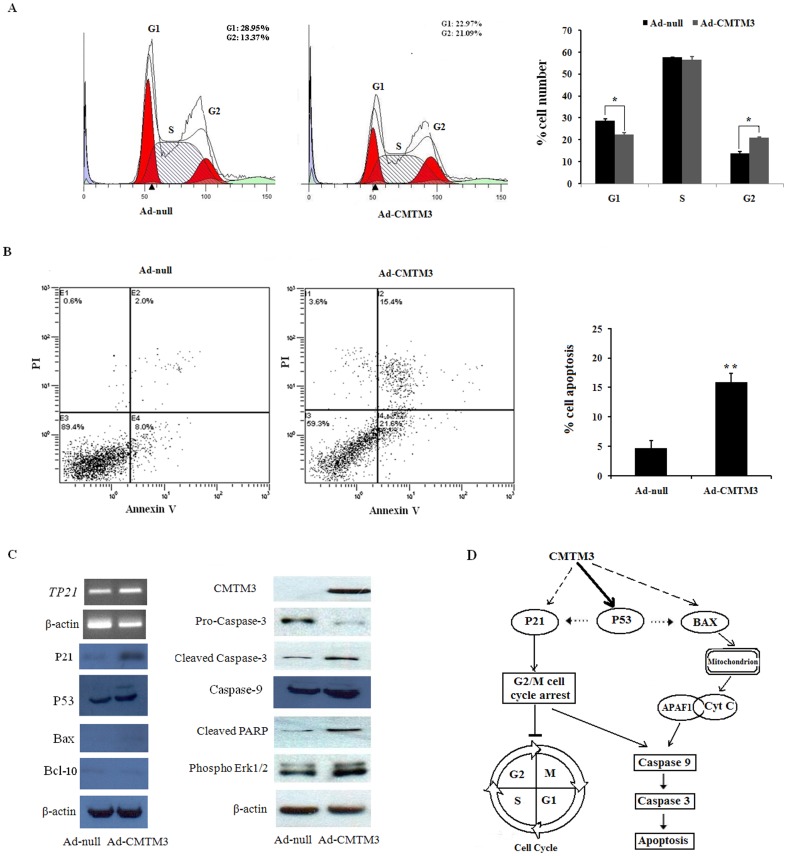
CMTM3 induces cell cycle arrest and apoptosis of NCCIT cells in a p53-independent manner. (A) Representative cell cycle analysis. The results are represented as the average ± S.D and are based on three independent experiments (p<0.01). G1 and G2 phases in red, S phases in white. (B) NCCIT cells infected with Ad-null and Ad-*CMTM3* were labeled with FITC-Annexin V/PI, and apoptosis was assessed by flow cytometry. (C) Western blot analysis of cell cycle- and apoptosis-related proteins in Ad-null- and Ad-*CMTM3*-infected NCCIT cells. β-actin was used as a control. All experiments were performed three times. (D) A schematic illustration of the proposed model depicting a p53-independent apoptosis pathway in testicular tumors.

The marked suppression of proliferation by CMTM3 prompted us to evaluate the effects of CMTM3 on the cell cycle distribution and apoptosis of testicular cancer cells. Representative results of the cell-cycle distribution in Ad-null- or Ad-*CMTM3*-infected NCCIT cells are shown in Figure 3A. Flow cytometry analysis revealed a significant G2 cell cycle arrest in CMTM3-expressing cells compared to the control-infected cells (Figure 3A).

We also investigated the apoptotic effect of CMTM3 in testicular cancer cells using Annexin V-FITC/PI staining assays. The proportion of Annexin V-positive/PI-positive cells increased by 15.4% in Ad-*CMTM3*-transduced NCCIT cells (Figure 3B). These results indicate that the inhibitory effect on cell proliferation by CMTM3 is most likely mediated by G2 cell cycle arrest and apoptosis.

### CMTM3 induces cell cycle arrest and apoptosis in a p53-independent manner

To explore the molecular mechanism of CMTM3-induced cell cycle arrest, we assessed the effect of CMTM3 re-expression on the expression of the cell cycle regulator, p21. As shown in Figure 3C, CMTM3 can increase the mRNA and protein levels of p21.

To determine the molecular mechanism of CMTM3-induced apoptosis, we utilized the Human Apoptosis RT^2^ Profiler PCR Array, which contains 84 known apoptotic genes, to monitor the expression profiles of NCCIT cells infected with Ad-*CMTM3*. To our surprise, the results showed that *TP53* and a series of genes (*APAF1*, *BAX*, and *BCL10*) were significantly up-regulated (Table 2). These results were verified by qPCR with different primers than those used in the PCR array. The protein levels were also increased by a similar magnitude (Figure 3C, Table 2).

**Table 2:** Summary of apoptosis-related genes significantly increased in Ad-*CMTM3*-infected NCCIT cells.

Gene symbol	Description	Function	RNA change^a^^,^ b	Protein change
*AIFM1*	apoptosis-inducing factor, mitochondrion-associated, 1	Pro/antiapoptosis	1.71	/
*APAF1*	apoptotic peptidase activating factor 1	Pro-apoptosis	1.51	/
*TP53*	tumor protein p53	Pro-apoptosis	141.75	3.22
*BAX*	BCL2-associated X protein	Pro-apoptosis	2.27	2.12
*BCL10*	B-cell CLL/lymphoma 10	Pro-apoptosis	1.69	1.53
*TP73*	Tumor protein 73	Pro/antiapoptosis	2.12	/

a Genes identified as increased 1.5-fold are listed.

b Fold change represents the ratio of signal in Ad-*CMTM3*-infected NCCIT cells relative to Ad-*null*-infected NCCIT cells for each primer set.

Next, we explored the activation of the apoptotic pathway by Western analysis. As shown in Figure 3C, the cleaved caspase-3 fragment was markedly increased, while pro-caspase-3 was markedly reduced in CMTM3 re-expressing testicular cancer cells compared with controls (Figure 3C). Re-expressing CMTM3 also markedly promoted caspase-9 protein expression in testicular cancer cells. Meanwhile, increased cleaved poly (ADP-ribose) polymerase was observed in CMTM3-transfected cells but was rarely detected in control cells (Figure 3C). These data suggest that CMTM3 facilitates testicular cancer cell apoptosis in a p53-independent manner, as p53 is not fully functional in NCCIT cells [24] (Figure 3D).

### Methylation of a specific, single CpG site in clinical testicular cancer specimens is significantly associated with CMTM3 transcript expression

As epigenetic regulation is involved in the expression of *CMTM3* [8], we asked whether *CMTM3* promoter DNA methylation is associated with a corresponding reduction in CMTM3 expression in testicular cancers.

**Figure 4:**
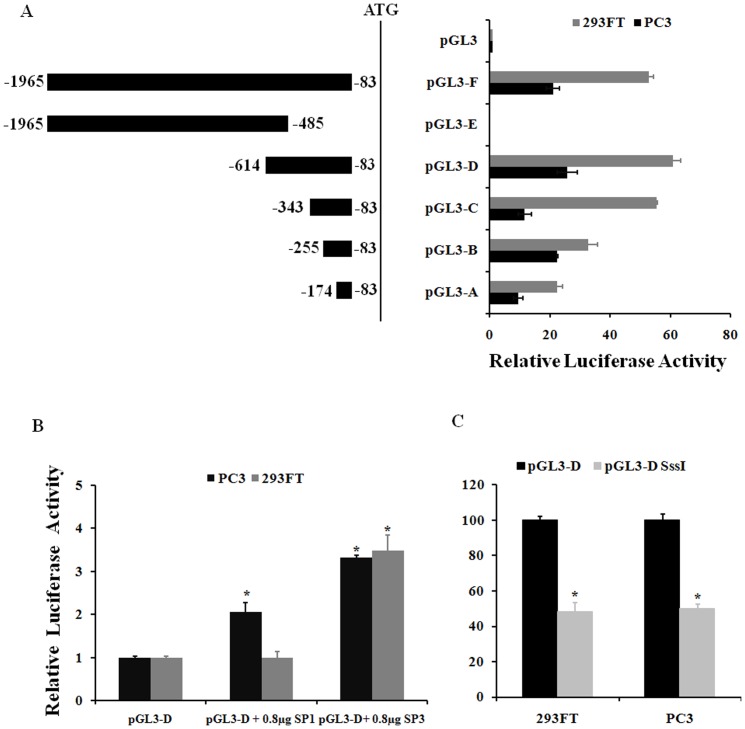
Promoter activity of human CMTM3 is regulated by Sp1/Sp3 and promoter methylation. (A) Promoter activity of human CMTM3 deletion constructs in 293FT and PC3 cells. The cells were transfected with 0.8 µg pGL3-basic or luciferase reporter constructs containing different size promoter fragments. (B) Effects of Sp1/Sp3 on reporter gene expression. 293FT and PC3 cells were transfected with pCMV-*Sp1/Sp3* expression plasmids or the empty vector as a control. (C) The promoter fragment (pGL-D) and the in vitro methylated fragment (pGL-PD SssI) were transfected into 293FT and PC3 cells, and a luciferase assay was performed. All constructs were cotransfected with pRLSV40 vector as an internal control for transfection efficiency. Luciferase activity is expressed as the percent activity of the control. The data are expressed as the means ± S.E.

To define the promoter region controlling *CMTM3* expression, a series of truncated promoter constructs were generated and inserted into the pGL3 luciferase reporter vectors, and the activity of the promoter was determined by assaying for luciferase. As shown in Figure 4A, the most significant promoter activity was found in a ∼400 bp fragment (−343 to −83 bp from start the codon site). Two putative binding sites for Sp1/Sp3 elements in the ∼400 bp fragment were identified by the transcription factor motif prediction software programs.

To confirm whether the relevant *cis*-acting elements, Sp1 and Sp3, are involved in the regulation of *CMTM3* gene expression, 293FT and PC3 cells were co-transfected with the construct pGL3-*PD* and pCMV-*Sp1* or pCMV-*Sp3*. Luciferase reporter gene assays showed that the overexpression of Sp1 or Sp3 could significantly increase *CMTM3* promoter activity (Figure 4B). Moreover, CMTM3 promoter activity was reduced dramatically after methylation (Figure 4C).

**Figure 5:**
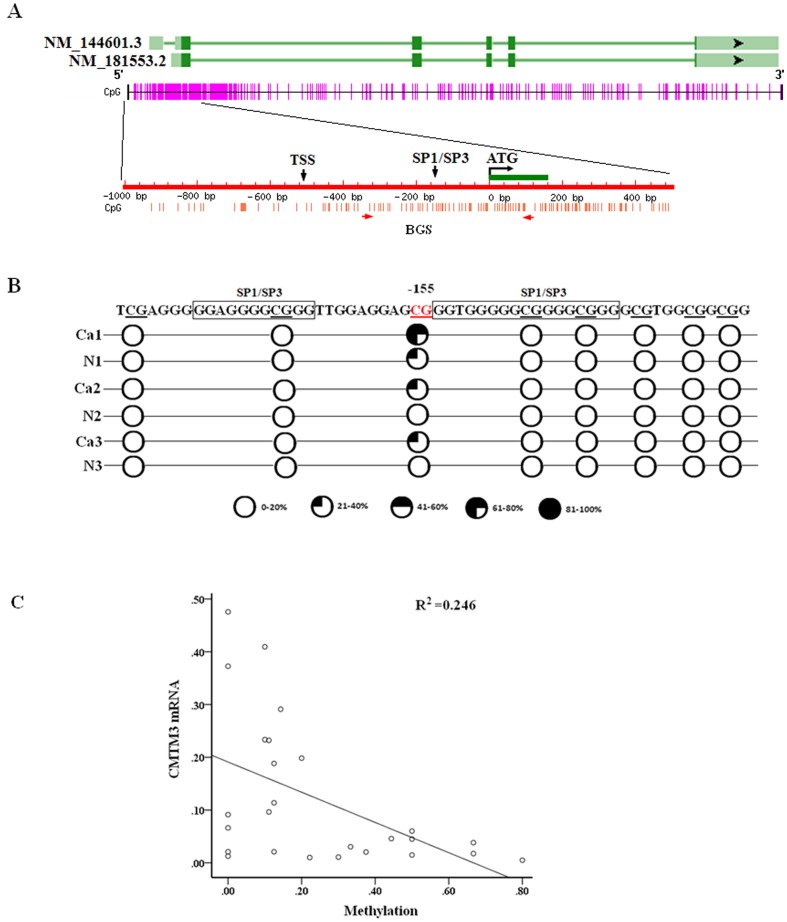
Expression of CMTM3 is silenced by the methylation of a single CpG site in the CMTM3 promoter region in testis cancer. (A) Schematic structure of the CMTM3 gene, CpG islands, putative transcription factor binding sites and the transcription start site. Exons are indicated by green rectangles. The translation start site is indicated by a curved arrow. The downward arrow indicates the potential binding site for Sp1/Sp3. BGS primers for methylation analyses are indicated. (B) The methylation of the CMTM3 promoter region was analyzed by BGS. The methylation of 8 CpG sites in the core promoter region for 3 paired tumor and corresponding benign adjacent tissues is shown. (C) The methylation of the single CpG site at −155 bp of the CMTM3 promoter region for 13 paired tumor and corresponding benign adjacent tissues, plotted as the CMTM3 mRNA expression in testicular cancer tissues. A statistically significant negative correlation is observed. Pearson correlation, p = 0.01, R^2^ = 0.246.

We next performed the bisulfite sequencing of 53 CpG sites including the *CMTM3* core promoter region (−353 to +126 bp from start the codon site) in 13 paired primary testis tumor samples (Figure 5A). The results showed that a single CpG site at −155 bp, located at the junction of the two Sp1/Sp3 binding sites, was more significantly methylated in tumor tissues (40.4% and 9.0%, respectively), in which *CMTM3* was down-regulated, than in corresponding non-tumor tissues (Figure 5B). No significant DNA methylation was observed at the other 52 CpG sites. A statistically significant inverse association was observed between the methylation of the single CpG site at −155 bp and the level of *CMTM3* mRNA expression in clinical samples (*P* = 0.01, R^2^ = 0.246) (Figure 5C). This finding suggests that *CMTM3* transcription in testicular cancer is determined, at least partially, by the methylation of a specific, single CpG site within the *CMTM3* promoter region.

## Discussion

*CMTM3* is highly expressed in normal testes [25], but its role in testicular cancer remains elusive. In the present study, we found that *CMTM3* was frequently downregulated or silenced in testicular cancer cell lines and primary tumors (Figure 1, Table 1).We further performed functional studies in NCCIT cells to unveil the biological function of CMTM3. We found that the re-expression of CMTM3 strongly impaired NCCIT cell motility and suppressed colony formation (Figure 2), which was consistent with previous reports in other cancers [8], [13]. Cell cycle analysis demonstrated that CMTM3 inhibited cell proliferation by increasing the proportion of cells in G2 phase and promoting cell apoptosis (Figure 3A, 3B). These data suggested that CMTM3 functions as a tumor suppressor in TGCTs. Previous studies showed that CMTM family proteins, including CMTM3 [8], CMTM5 [26], [27], and CMTM8 [28], [29], can induce cell apoptosis. However, the apoptotic pathway and the mechanism remain elusive. In the present study, we found that the re-expression of CMTM3 in NCCIT cells promoted the transcription of *TP53*, *P21*, and *BAX*, and increased their protein levels by a similar magnitude (Table 2, Figure 3C). p53 is not fully functional in NCCIT cells, suggesting that there is no clear correlation between the trans-activation of p53 and the induction of apoptosis in NCCIT cells [24]. However, p21 was significantly increased. Thus, the possibility was raised that CMTM3 could induce a G2 cell cycle arrest and facilitate cell apoptosis through p21 or other pathways in a p53-independent manner, as reported in other studies (Figure 3D) [30]–[32].

The down-regulation of tumor suppressors is associated with transcriptional inhibition through the induction of repressive epigenetic modifications in the promoter, including DNA methylation and histone modification [33]. Previous studies showed that CMTM family genes *CMTM3* and *CMTM5* are silenced or down-regulated by promoter CpG methylation in several carcinoma cell lines and primary tumors [8], [9], [13], [34]. Our present study shows that the CpG sites near the core promoter of the CMTM3 gene are nearly devoid of methylation in TGCTs (Figure 5A, 5B), which supports the notion that the aberrant de novo methylation of tumor suppressor genes or tumor-related genes is a rare event in TGCTs [35]. However, a single CpG site located at the junction of the two Sp1/Sp3 binding sites had moderate methylation, and the methylation values were significantly inversely correlated with the expression of *CMTM3* in tumor tissues (Figure 5C). This result suggested that the expression of *CMTM3* in TGCTs is regulated, at least partially, by the methylation status of the single CpG site at −155 bp. A similar phenomenon was observed in other recent studies, in which a handful of single CpG islands were identified to be essential for gene regulation via methylation [36]–[42]. It seems plausible that the methylation of the single CpG site can obstruct the binding of Sp1/Sp3, thus affecting the recruitment of Sp1/Sp3-associated regulatory proteins to the promoter of the gene, as was observed in other studies [43]–[46].

Collectively, *CMTM3* is frequently down-regulated in TGCT tissues, and the reduction of CMTM3 protein is associated with advanced tumor stage. CMTM3 is a potential tumor suppressor gene in TGCTs that functions by inhibiting cell proliferation and inducing apoptosis in a p53-independent manner. The transcriptional repression of CMTM3 in TGCTs may be mediated by the methylation of a single CpG site, even if the overall methylation of the genes is very limited, which would suggest that the underlying epigenetic mechanisms are different between TGCTs and cancer cells of somatic tissue origin [47]. Further investigations are warranted to bolster the present conclusion due to the limited testicular cancer tissue samples available.

## Supporting Information

File S1 **Figure S1, Lack of detectable CMTM3 protein expression is frequent in testis cancer tissues. Table S1, Primers for a series of truncated CMTM3 promoter constructs. Table S2, Correlation between CMTM3 expression in seminoma with pathological parameters.**
(DOC)
