# Supplementary material for: CMTM3 Inhibits Human Testicular Cancer Cell Growth through Inducing Cell-Cycle Arrest and Apoptosis
Source: PLoS One. 2014 Feb 28;9(2):e88965. doi: 10.1371/journal.pone.0088965 (PMC3938458; doi:10.1371/journal.pone.0088965)
Supplement: File S1 — Figure S1, Lack of detectable CMTM3 protein expression is frequent in testis cancer tissues. Table S1, Primers for a series of truncated CMTM3 promoter constructs. Table S2, Correlation between CMTM3 expression in seminoma with pathological parameters. (DOC) [file pone.0088965.s001.doc]

**Table S1. Primers for a series of truncated CMTM3 promoter constructs**

| No. | Name | Primer (5’-3’) | Product |
| --- | --- | --- | --- |
| 1 | A-F | ATAGGTACCGAGGGGCGGGCTGGAGGA | 91bp |
| 2 | B-F | ATAGGTACC AGAAGGTGGAGTCTGCGGAGG | 166bp |
| 3 | C-F | ATAGGTACC TTCTGGATAGGGGCAGACGG | 261bp |
| 4 | D-F | ATAGGTACC TCCTCTGAGAATGGGTTGGGG | 532bp |
| 5 | A,B,C,D-R | GACAGATCT GAAGGAGGGCAGGCGACAC |  |
| 6 | E,F-F | ATAGGTACC CCAGACCCAAGGAGCCAATT |  |
| 7 | E-R | GACAGATCT CGGAGTTTTCTTGCGACTTTCT | 1481bp |
| 8 | F-R | GACAGATCT GAAGGAGGGCAGGCGACAC | 1883bp |

| **Table S2. Correlation between CMTM3 expression in seminoma with pathological parameters** | | | | | |
| --- | --- | --- | --- | --- | --- |
| Clinical-pathologic variables | No of Case | CMTM3 expression | | χ^2^ | p |
|  |  | Negative | Positive |  |  |
| **All cases** | **46** | **25** | **21** |  |  |
| **Ages** |  |  |  |  |  |
| >45 | 21 | 16 | 16 | 0.371 | 0.522 |
| <45 | 25 | 9 | 5 |  |  |
| **T stage** |  |  |  |  |  |
| T1 | 25 | 9 | 16 | 7.41 | 0.024 |
| T2 | 16 | 12 | 4 |  |  |
| T4 | 5 | 4 | 1 |  |  |

**Figure S1.** Lack of detectable CMTM3 protein expression is frequent in testis cancer tissues. A tissue microarray containing 101 tissue samples from normal testis and the various histologic subgroups of TGCT was used. Representative examples of CMTM3 immunohistochemical (IHC) staining in the various histologic subgroups of testis cancer and normal testis tissues are shown. (A) Seminoma. (B) Yolk sac tumor. (C) Embryonal carcinoma. (D) Cancer adjacent normal tissue.

**A B**

**
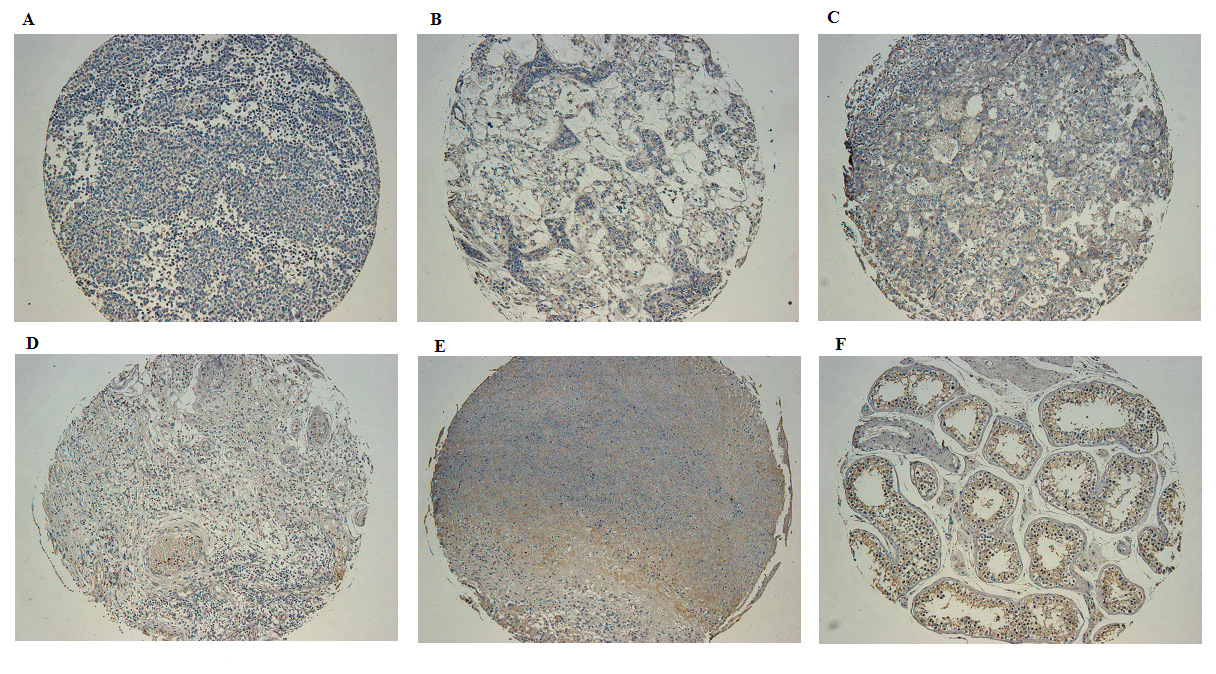
**

**C D**

**
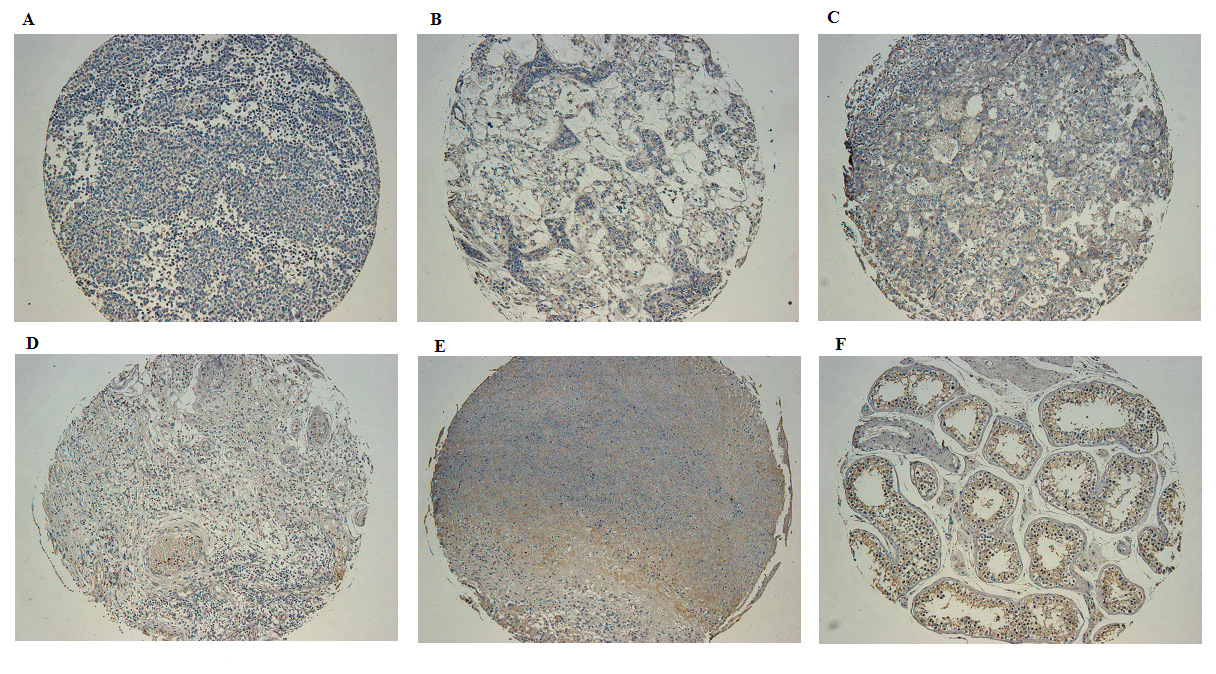

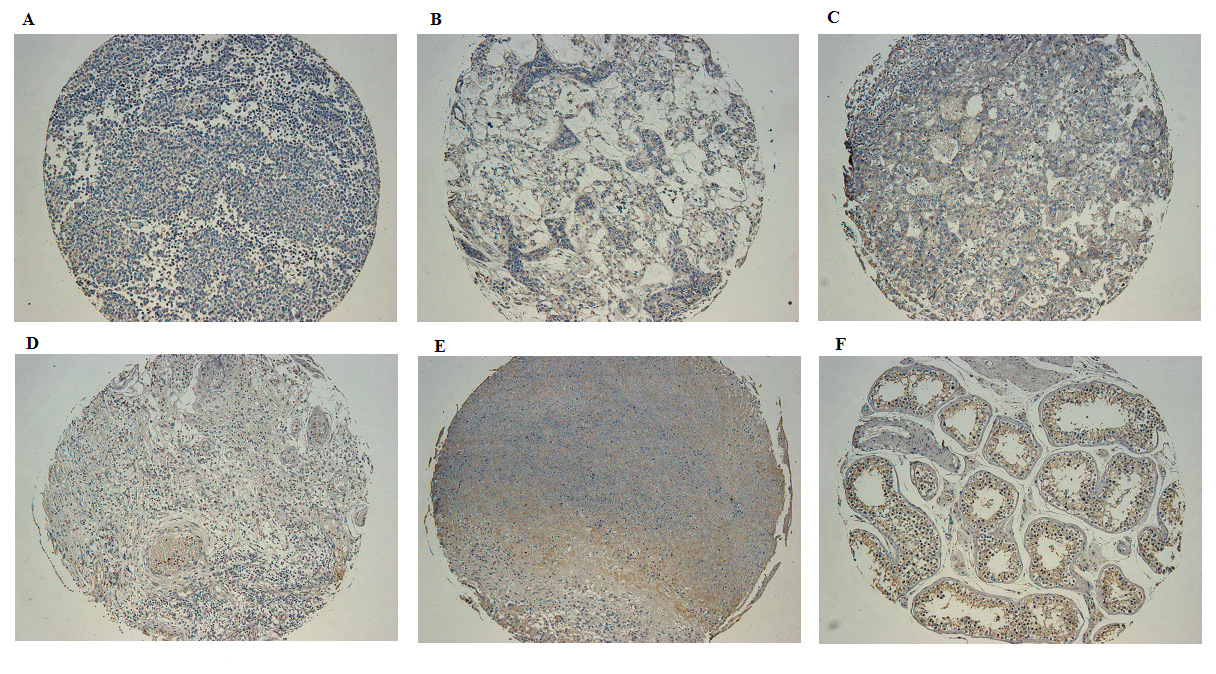
**
